# Impact of neurite alignment on organelle motion

**DOI:** 10.1098/rsif.2021.0617

**Published:** 2022-02-09

**Authors:** Maria Mytiliniou, Joeri A. J. Wondergem, Thomas Schmidt, Doris Heinrich

**Affiliations:** ^1^ Leiden Institute of Physics, Huygens-Kamerlingh Onnes Laboratory, Leiden University, 2333 CA Leiden, The Netherlands; ^2^ Institute for Bioprocessing and Analytical Measurement Techniques, Rosenhof, 37308 Heilbad Heiligenstadt, Germany; ^3^ Faculty for Mathematics and Natural Sciences, Technische Universität Ilmenau, 98693 Ilmenau, Germany; ^4^ Fraunhofer Institute for Silicate Research ISC, 97082 Würzburg, Germany

**Keywords:** intracellular dynamics, lysosomes, neurite alignment, microscopy, motion analysis

## Abstract

Intracellular transport is pivotal for cell growth and survival. Malfunctions in this process have been associated with devastating neurodegenerative diseases, highlighting the need for a deeper understanding of the mechanisms involved. Here, we use an experimental methodology that leads neurites of differentiated PC12 cells into either one of two configurations: a one-dimensional configuration, where the neurites align along lines, or a two-dimensional configuration, where the neurites adopt a random orientation and shape on a flat substrate. We subsequently monitored the motion of functional organelles, the lysosomes, inside the neurites. Implementing a time-resolved analysis of the mean-squared displacement, we quantitatively characterized distinct motion modes of the lysosomes. Our results indicate that neurite alignment gives rise to faster diffusive and super-diffusive lysosomal motion than the situation in which the neurites are randomly oriented. After inducing lysosome swelling through an osmotic challenge by sucrose, we confirmed the predicted slowdown in diffusive mobility. Surprisingly, we found that the swelling-induced mobility change affected each of the (sub-/super-)diffusive motion modes differently and depended on the alignment configuration of the neurites. Our findings imply that intracellular transport is significantly and robustly dependent on cell morphology, which might in part be controlled by the extracellular matrix.

## 1. Introduction

One critical function—not only for the growth and maintenance of homeostasis but also for the survival of a cell—is the transport of proteins, molecules, organelles and debris to specific locations within the cell. This allocation is of tremendous significance, especially for neuronal cells owing to the extreme size of their axons; for instance, axons of human motor neurons can reach a length of 1 m, starting from the brain and extending as far as the end of the spine. Defects in the process of intracellular transport have long been associated with human diseases [1–5], but whether such defects are the cause or the consequence of pathological phenotypes is still under debate in several cases [6,7].

Intracellular distribution of molecules and organelles is achieved mainly via two mechanisms: passive diffusion and active, motor-driven transport along microtubules and actin filaments [8]. Cytoskeletal components, organelles and molecules crowd the cytoplasm, thereby hindering or enhancing passive diffusion, thus leading to sub-diffusive and super-diffusive intracellular motion [9–12]. In order to characterize intracellular dynamics and extract values such as the velocity of the motor-mediated transport or the diffusion coefficient of passive motion, several models have been implemented [13–15].

A frequently used approach exploits the mean squared displacement (MSD), plotted as a function of lag time and subsequently fitted with a power law in the form of ∼2*dDτ*^*α*^, where *d* is the dimensionality, *D* is the diffusion coefficient and *τ* is the lag time [16,17]. The characteristic exponent (*α*) value reveals the type of motion, differentiating among Brownian diffusion (*α* = 1), from now on referred to as diffusion, super-diffusion (*α* > 1) and sub-diffusion (*α* < 1). In the case of complex motion, such as intracellular transport, comprising a combination of alternating phases of sub-diffusion, free diffusion and motor-driven active transport, motion-discriminating algorithms are essential to avoid averaging out dynamic information. Thus, local-MSD (lMSD) analysis can be performed instead of fitting the entire MSD curve, providing time-resolved information about the motion states within a trajectory. This analysis implements a rolling window over the entire trajectory, thereby characterizing each data point with an *α* exponent value [9,10,18–20].

Although the MSD analysis is a well-established tool, a reliable and less noisy MSD curve requires many data points and long trajectories, which, in the case of biological data, can be challenging to acquire [21]. Additionally, selection of the model to fit is not always straightforward, especially in the instance of complex data with multiple underlying processes [22]. Furthermore, the MSD curve is sensitive to experimental parameters such as the acquisition frame rate and the size of the imaged particle [16].

Hence, the use of an additional method, the van Hove distribution [23], for the analysis of intracellular data can counterweigh the drawbacks described above and provide a more in-depth view of intracellular transport than that gained from the MSD analysis alone. The van Hove distribution, its self-part is alternatively called the jump distance distribution (JDD), was initially used for particle-scattering experiments [24,25]. The JDD is plotted for a specific lag time and depicts the Euclidean displacement distribution of the observed particles within the given time lag, in the form of a probability distribution function (PDF). Thus, this distribution reveals the displacement-dependent structure that is, otherwise, ‘hidden’ in a single, averaged data point, for the respective lag time, of the MSD curve.

In 1997, Schütz *et al.* showed that, by fitting the probability distribution of the squared displacements of single molecules moving on membranes, one can extract individual diffusion constants and fractions of multiple-component samples [26]. Along the same lines, Kues *et al.* analysed single-molecule motion inside cell nuclei, distinguishing among three mobility states [27]. Over time, application of this analysis for Brownian motion in fluids [28] and in—actual or simulated—biological systems increased [11,17,29–31], establishing it as a powerful tool for the characterization of complex biological trajectories.

Here, we set out to characterize trajectories of lysosomes inside neurites of differentiated PC12 cells [32–34], commonly used as a neuronal model [35–37]. Neurites are the precursors of dendrites and axons in immature neurons [38]; hence, motion analysis within neurites can provide significant insight into axonal transport. Lysosomes are organelles that play a vital role in the autophagy pathway of cells and exhibit both diffusive motion and active transport via dynein and kinesin motor proteins along microtubules. Both the autophagy pathway in general [39–41] and the motion of lysosomes specifically appear to be implicated in neurodegenerative diseases and cancer [42–45].

We show that neurite alignment, achieved via chemical surface patterning, results in faster diffusive and super-diffusive lysosomal motion than the case in which neurites adopt a random orientation. Moreover, we introduce a perturbation in the cellular environment via incubation with sucrose and confirm experimentally that the sucrose induces lysosomal enlargement, which leads to a proportionate decrease in the diffusion coefficient. Implementing lMSD analysis, we identify and extract the trajectory parts that belong to each of three classes of motion, namely sub-diffusive, diffusive and super-diffusive. By collectively analysing the data points of the respective class, we gain quantitative insights for each motion mode. Our findings indicate that incubation with sucrose results in a different effect on each motion mode of the organelles, and this also depends on the configuration of the neurites within which the motion occurs.

## 2. Methods

### 2.1. Laminin μPIP

The mould for the patterning mask was fabricated with a Nanoscribe Photonic professional GT 3D laser printer (Nanoscribe, Germany), with two-photon polymerization (2PP) of IP-S photoresist [46]. Prior to the first use and after each subsequent use, a layer of trichloro(1*H*,1*H*,2*H*,2*H*-perfluorooctyl)silane (Sigma-Aldrich) was deposited on the silicon mould (silanization) to reduce stiction [47].

Polydimethyl-siloxane (PDMS; Sylgard 184, Dow Corning, USA) was prepared by mixing the cross-linking agent with the elastomer base at a ratio of 1 : 10. The mixture was pipetted onto the silicon mould and allowed to cross-link for 1 h at 120°C. Subsequently, the hardened PDMS bearing the structure was peeled from the wafer.

For plasma-initiated patterning (PIP), the PDMS mask was placed on an Ibidi dish (Ibidi GMBH; μ-Dish; 35 mm high, polymer coverslip bottom), with the structure-bearing side adherent to the bottom of the dish, and was exposed to air plasma for 6 min 20 s at 100 W (Diener Electronic Femto Plasma system).

Subsequently, the PDMS mask was removed and the substrate was flooded with 0.1% Pluronic F127 (Sigma-Aldrich) diluted in phosphate-buffered saline (PBS) for 45 min at room temperature. The dish was then washed three times with PBS and once with RPMI (Gibco^TM^) and subsequently incubated with $25\;\mu g\;ml^{-1}$ laminin (Sigma-Aldrich) diluted in RPMI for 1 h at 37°C. Prior to seeding cells, the substrate was washed three times with RPMI.

### 2.2. Cell culture

PC12 cells (CH3 BioSystems) were cultured in dishes coated with rat-tail collagen (CH3 BioSystems). Their growth medium consisted of 85% RPMI-1640 with Glutamax (Gibco^TM^), 10% heat-inactivated horse serum (HS) (Sigma-Aldrich), 5% heat-inactivated fetal calf serum (FCS HI; Thermo Scientific) and 200 μg ml^−1^ penicillin/streptomycin (PS). The medium was refreshed three times per week, and the cells were split once per week at a ratio of 1 : 3–1 : 6. The cells were kept at 37°C and 5% CO_2_ under a humidified atmosphere.

To induce differentiation, PC12 cells were seeded in Ibidi uncoated dishes coated with laminin. For whole-surface laminin coating, the dish was exposed to air plasma for 6 min 20 s at 100 W (Diener Electronic Femto Plasma system). Subsequently, the dish was incubated for 1 h at 37°C and 5% CO_2_ with 25 μg ml^−1^ laminin diluted in RPMI.

Cells were seeded at a density of 30 000 cells/cm^2^ in full medium; after they had adhered, they were washed once with PBS and then the medium was replaced with differentiation medium consisting of Opti-MEM^TM^ reduced serum medium (Gibco^TM^) supplemented with 0.5% fetal bovine serum (Gibco^TM^) and nerve growth factor (NGF-2.5S; Sigma-Aldrich) at a final concentration of 100 ng ml^−1^. The differentiation medium was refreshed three times per week.

To induce swelling of lysosomes, 50 mM sucrose (Sigma-Aldrich) was added in the differentiation medium 18 h before imaging. At the end of the incubation, the cells were washed once with PBS and submerged again in normal culture or differentiation medium.

Prior to imaging, the cells were incubated with 50–150 nM Lysotracker (Invitrogen^TM^) in RPMI for 30 min.

For immunofluorescent staining, differentiated PC12 cells were fixed with 4% paraformaldehyde for 15 min at room temperature, permeabilized with 0.1% TritonX for 10 min and blocked with 2% bovine serum albumin (BSA) in PBS for 60 min. Subsequently, the cells were incubated with *α*-tubulin antibody (Alexa Fluor^®^ 488 anti-alpha Tubulin antibody; Abcam) 1:150 in 0.1% BSA in PBS at 4°C for 18 h. Next, the cells were incubated with phalloidin antibody (iFluor 647; Abcam) 1 : 1000 in 0.1% BSA in PBS at room temperature for 60 min. Lastly, the cells were incubated for 30 min at room temperature with Hoechst (Invitrogen) diluted to 1 μg ml^−1^ in 1% BSA in PBS and stored in PBS at 4°C until imaging.

### 2.3. Optical microscopy

Optical microscopy images were acquired with a Nikon Ti Eclipse inverted microscope (Nikon Corporation, Japan) equipped with a Yokogawa CSU-X1 spinning disc unit (10 000 r.p.m.; Andor Technology Ltd, UK). The samples were imaged with a 100× objective (Nikon CFI Plan Apo Lamda; NA 1.45). Excitation at 405 nm, 488 nm and 647 nm was achieved via an Agilent MLC400 monolithic laser combiner (Agilent Technologies, The Netherlands). The excitation light was filtered by a custom-made Semrock quad-band dichroic mirror for excitation wavelengths of 400–410, 486–491, 460–570 and 633–647 nm. The emitted light was filtered using a Semrock quad-band fluorescence filter (TR-F440-521-607-700), which has specific transmission bands at 440 ± 40 nm, 521 ± 21 nm, 607 ± 34 nm and 700 ± 45 nm and by Semrock Brightline single-band fluorescence filters at 447 ± 60 nm (TR-F447-060) and 525 ± 60 nm (TR-F525-030). Images were captured with an Andor iXon Ultra 897 high-speed EM-CCD camera. Image acquisition was automated using NisElements software (LIM, Czech Republic). Time-lapse images were acquired every 18 ms, for up to 30 s. During data acquisition, the cells were kept under a humidified atmosphere at 37°C and supplied with 5% CO_2_ via the use of a Tokai Hit stage incubator.

### 2.4. Data analysis

All analysis was performed for lysosomes located at various points within the neurites, and thus at varying distances from the cell body, without differentiating among specific regions of the neurites or directionality of the lysosomal motion.

The Feret diameter of lysosomes was calculated using FIJI [48]. The selection of lysosomes in untreated cells was performed using an area mask between 0.1 μm^2^ and 1.5 μm^2^. Lysosomes in cells that had been treated with sucrose were selected with a mask area of between 0.5 μm^2^ and 5.0 μm^2^. Threshold values were the same for all images and clustered lysosomes were discarded from the analysis.

Trajectories of fluorescent lysosomes were tracked using the FIJI plugin, TrackMate [49], which returned the *x*- and *y*-coordinates of the centre of the lysosomes, with a sub-pixel localization. During the tracking, we included all three populations of lysosomes: those that appeared to be confined, diffusive and motile, with bigger (motor-mediated) displacements.

Further processing was performed using home-made Matlab algorithms. The *x*- and *y*-coordinates as a function of time for each trajectory were represented by a series of vectors at each time point *t*,

2.1 r(t)=x(t)+y(t),

and the displacement Δ**r** at time *t* was calculated as follows:

2.2 Δr(t)=r(t+Δt)−r(t),

where Δ*t* is the inverse frame rate.

The MSD for lag time *τ* = *k*Δ*t* was calculated according to

2.3 $$\mathrm{MSD}(\tau )=\left\langle \Delta r^{2}(\tau )\right\rangle =\frac{1}{N-k}\sum_{i=1}^{N-k}{\left(r(t_{i}+\tau )-r(t_{i})\right)}^{2},$$

where *N* is the number of data points in the trajectory and *k* = 1, 2, …, *N* − 1. The average MSD per condition is the average of the squared displacements of all lysosome trajectories for each lag time *τ*.

The lMSD was calculated for each trajectory as described previously [18]. Briefly, the MSD was calculated for each data point of the entire trajectory using a rolling window of 2.22 s (*N* = 120, in equation (2.3)) and fitted for the interval 0–555 ms (*k* = 30 in equation (2.3)) with a power law

2.4 $$\mathrm{MSD}(\tau )=A\tau^{\alpha }.$$

The alpha exponent as a function of time was subsequently used to partition the transport states as sub-diffusive for *α* < 0.9, diffusive for 0.9 ≤ *α* ≤ 1.1 or super-diffusive for *α* > 1.1.

In order to characterize more closely each type of motion, we analysed collectively the respective trajectory parts, for each experimental condition (cells in medium without (control) or with sucrose and in neurites that were aligned or randomly oriented).

The average MSD curve for each motion mode was calculated again according to equation (2.3). The sub-diffusive trajectory modes MSD was fitted with the power law describing anomalous diffusion

2.5 $$\mathrm{MSD}(\tau )=A\tau^{\alpha }+2d\sigma^{2},$$

thereby obtaining the value of the anomalous *α* exponent (*A* is a constant). The MSD curve was fitted for all lag times (up to 30 s).

The diffusive trajectory modes MSD was fitted using the equation describing Brownian motion

2.6 $$\mathrm{MSD}(\tau )=2dD\tau +2d\sigma^{2},$$

thus extracting the experimental value of the diffusion coefficient *D*. The MSD curve was fitted for lag times 1–10 (18.5–185 ms).

The super-diffusive trajectory modes MSD was fitted using the model of Brownian motion with drift [50],

2.7 $$\mathrm{MSD}(\tau )=2dD_{\mathrm{eff}}\tau +V_{\mathrm{drift}}^{2}\tau^{2}+2d\sigma^{2},$$

where *V*_drift_, the constant drift parameter, models the velocity of the molecular motors. The MSD curve was fitted for lag times 1–55 s (0.0185–1 s).

In equations (2.5)–(2.7) *σ* is the localization precision, *τ* is the lag time and the parameter *d* refers to the dimensionality. *d* was set equal to 1 for the fit of the MSD along the *x*- or *y*-axes, and equal to 2 for the fit of the two-dimensional MSD curve.

The JDD was calculated according to the self-part of the van Hove correlation function [23],

2.8 $$G_{s}(\Delta r,\tau )=\frac{1}{k}\sum_{i=1}^{k}\left\langle \delta (\Delta r-r(t_{i}+\tau )+r(t_{i}))\right\rangle ,$$

for displacements in both the *x*- and *y*-directions, and a specific lag time *τ*. *δ* here denotes the Dirac delta function in two dimensions and *k* = 1, 2, …, *N* − 1, with *N* the number of data points in the trajectory. The bin size was (arbitrarily) set to 1 μm and the JDD was normalized into a PDF.

The diffusive and super-diffusive trajectory parts for each experimental condition were used to calculate the respective JDD PDF, for lag times of 0.2405 ms and 0.7585 ms, respectively. The PDFs were fitted to extract characteristic values of the motion, using the analytical expressions calculated in [30]. Particularly, the diffusive trajectory modes JDD PDF was fitted for the *x*-direction, using

2.9 $${\mathrm{JDD}}_{\mathrm{PDF}}=\frac{1}{\sqrt{\pi D\tau }}\exp\left(\frac{-x^{2}}{4D\tau }\right),$$

thereby estimating the experimental value of the diffusion coefficient *D*. Similarly, for the *y*-direction. The super-diffusive trajectory modes JDD PDF was fitted for the *x*-direction, using

2.10 $$\begin{array}{r} {\mathrm{JDD}}_{\mathrm{PDF}}=\frac{1}{\sqrt{4\pi D_{\mathrm{eff}}\tau }}\exp\left(\frac{-x^{2}+V_{\mathrm{drift}}^{2}\tau^{2}}{4D_{\mathrm{eff}}\tau }\right)\\ \left(\exp\left(\frac{V_{\mathrm{drift}}x}{2D_{\mathrm{eff}}}\right)+\exp\left(\frac{-V_{\mathrm{drift}}x}{2D_{\mathrm{eff}}}\right)\right) \end{array},$$

estimating the experimental value of the drift velocity *V*_drift_ and effective diffusion coefficient *D*_eff_. Likewise for the *y*-direction.

The coherency coefficients of microtubules and actin filaments within neurites were estimated using the OrientationJ FIJI plugin (coefficient values close to zero represent no preferential orientation, whereas values close to 1 correspond to strongly coherent orientation) [51].

## 3. Results

We set out to characterize intracellular organelle transport and to compare motion features under two distinct neurite configurations. We investigated the motion of lysosomes inside neurites of differentiated PC12 cells, when those adopt a random orientation on the two-dimensional culture surface, versus when they are prompted to adhere to a one-dimensional configuration by means of chemical surface patterning.

### 3.1. Microscale plasma-initiated patterning (μPIP) of laminin guides the neurites of differentiated PC12 cells along 2-μm-wide lines

**Figure 1.**
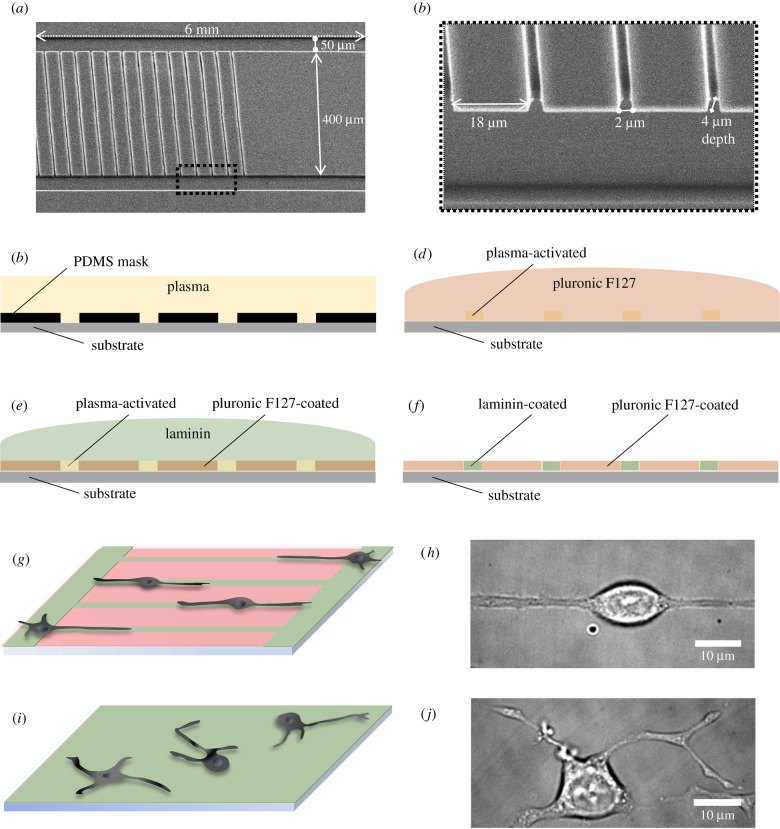
Laminin μPIP for neurite guidance. (*a*) SEM image of the PDMS mask used for the PIP. The structure consisted of two side lines, 50 μm wide and 6 mm long, with 400 μm distance in between. The central 2 mm of the 6 mm blocks was intersected by 2-μm-wide lines, repeated every 18 μm. The depth of the mask was 4 μm. (*b*) Close-up of the area indicated by the black dotted-line square in (*a*). (*c*–*f*) Steps followed for the μPIP. (*c*) The PDMS mask was placed on the substrate, and the assay was exposed to air plasma. (*d*) The PDMS mask was removed and the substrate was submerged in pluronic F127, which adsorbed to the plasma-protected areas. (*e*) The dish was immersed in laminin, which adhered to the plasma-activated areas. (*f*) The resulting pattern of laminin-coated lines surrounded by pluronic-covered regions. (*g*) Schematic of the patterned substrate. Green colour represents the ECM protein (laminin) and the red colour the pluronic F127. (*h*) Representative bright-field image of a differentiated PC12 cell on the patterned substrate, with its neurites aligned along the line. (*i*) Schematic of the unpatterned substrate, coated with laminin (green colour). (*j*) Representative bright-field image of a differentiated PC12 cell with the neurites randomly oriented in two dimensions.

We achieved one-dimensional neurite alignment by selective protein deposition on the cell substrate. The steps followed during the μPIP are schematically shown in figure 1*c*–*f*. A PDMS mask bearing a ladder-shaped pattern was used. Scanning electron microscopy (SEM) images of the PDMS mask are shown in figure 1*a*,*b*. The mask was inverted and pressed onto the cell substrate. The assay was then exposed to air plasma (figure 1*c*), thereby altering the surface charge of the areas exposed via the mask and thus increasing their hydrophilicity. The rest of the surface, covered by the adhered PDMS mask, remained in its original hydrophobic state. Subsequent incubation with pluronic F127 and laminin (figure 1*d*,*e*, respectively) resulted in 2-μm-wide laminin lines, alternating with 18-μm-wide pluronic F127-coated stripes (figure 1*f*).

In order to obtain the second neurite geometry, the entire substrate was coated with the extracellular matrix (ECM) protein. PC12 cells were allowed to adhere on both substrates, as schematically shown in figure 1*g*,*i*. Subsequently, the cells were differentiated to stimulate neurite growth (see Methods). Depending on the substrate used, patterned or unpatterned, the neurites either aligned along the lines or grew randomly on the two-dimensional surface. Representative cells of both configurations are shown in figure 1*h*,*j*, respectively.

### 3.2. Sucrose induces lysosomal enlargement in differentiated PC12 cells

Next, we wondered whether a perturbation in the cellular environment that associates with lysosomes could be deciphered by studying their motion and, if that was the case, whether the effect would differ for the two different neurite configurations. To investigate that question, we employed sucrose-induced swelling of lysosomes. Sucrose has long been known to trigger swelling of lysosomes; it enters the cytoplasm by pinocytosis but cannot be degraded by lysosomal enzymes, thus causing osmotic pressure alterations in lysosomes, which, in turn, by attempting to maintain osmotic balance, allow water influx, thus swelling [52]. Lysosome enlargement, along with its effect on lysosomal transport, has been quantified in BS-C-1 monkey kidney epithelial cells [53].

**Figure 2.**
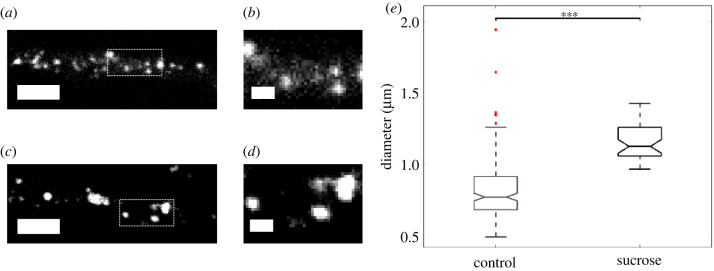
Sucrose induces an increase in lysosome diameter. (*a*,*b*) Fluorescent lysosomes of a cell in medium without sucrose (control). (*c*,*d*) Fluorescent lysosomes of a cell in medium with sucrose. Scale bar in (*a*,*c*) is 5 μm and the square indicates the location shown in the higher magnification in (*b*,*d*), respectively, with scale bar 1 μm. (*e*) Boxplot of lysosome diameters for differentiated PC12 cells in medium without sucrose (control) versus medium with sucrose, with mean values equal to 〈*d*_*c*_〉 = 0.82 ± 0.02 μm and 〈*d*_*s*_〉 = 1.15 ± 0.02 μm, respectively. The statistical significance between the two means was determined using the Wilcoxon ranksum test; *** corresponds to *p* < 0.001.

Along the same lines, we incubated the differentiated PC12 cells with sucrose prior to data acquisition. Representative images of fluorescent lysosomes inside differentiated PC12 cells in normal (control) medium and in medium containing sucrose are displayed in figure 2. A small effect on the size of the lysosomes can be observed by visual inspection. To quantify this, we measured the diameters of lysosomes for the two conditions; the distributions of the values are shown in figure 2*e*. The mean diameter was found to be equal to:

〈*d*_*c*_〉 = 0.82 ± 0.02 μm for lysosomes of cells in normal medium (control) and
〈*d*_*s*_〉 = 1.15 ± 0.02 μm for lysosomes of cells in sucrose-containing medium, resulting in an increase of 0.33 μm for the average lysosome diameter caused by incubation with sucrose.

### 3.3. Lysosomes inside aligned neurites exhibit higher displacements

**Figure 3.**
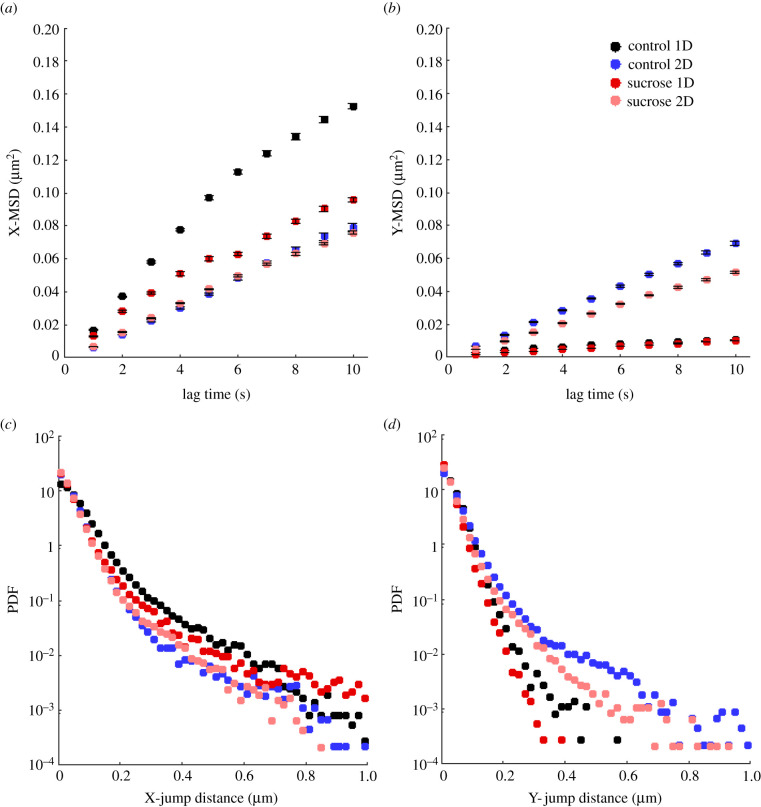
Larger MSD and JDD PDF values observed for lysosomes moving inside aligned neurites. (*a*) MSD curves along the *x*-axis and (*b*) MSD curves along the *y*-axis of lysosomal trajectories. Data points show time-averaged MSD ± standard error of the mean. (*c*) JDD PDFs along the *x*-axis and (*d*) JDD PDFs along the *y*-axis of lysosomal trajectories. Colour coding indicates lysosomes inside aligned (one-dimensional, 1D) or randomly oriented (two-dimensional, 2D) neurites of differentiated PC12 cells in medium without (control) or with sucrose.

Fluorescently labelled lysosomes were tracked for up to 30 s, inside both neurite configurations. The MSDs and JDD PDFs were calculated for all lysosomal trajectories of each condition (using equations (2.3) and (2.8), respectively), for the *x*- and *y*-displacements, and are displayed in figure 3. The *x*-axis coincided with the neurite alignment axis, in the corresponding experimental configuration.

As can be observed in figure 3*a*, the MSD curve along the *x*-axis exhibits significantly higher values for lysosomes inside aligned neurites, especially for the control condition. It is noteworthy that, even in the presence of the sucrose-induced perturbation, the *x*-MSD values of lysosomes inside aligned neurites are higher than those of the control condition in randomly oriented neurites. Moreover, the decrease in the displacement observed in the presence of sucrose is more significant for lysosomes inside aligned neurites than for those inside randomly oriented neurites. These findings are consistent with the JDD PDFs along the *x*-axis (figure 3*c*).

The MSD and JDD PDFs along the *y*-axis (figure 3*b*,*d*) clearly indicate the underlying neurite alignment of the corresponding experimental condition. On the other hand, the *y*-MSD and JDD PDF values of lysosomes inside randomly oriented neurites are similar to those for the *x*-axis. This result is expected since there is no directionality preference for lysosomes moving inside randomly oriented neurites.

### 3.4. Local MSD analysis of lysosome trajectories distinguishes among sub-diffusive, diffusive and super-diffusive motion modes

The shape of the MSD and JDD PDF curves presented in figure 3 indicates that the motion analysed here consists of more than one type of transport, as explained previously. To characterize each transport mode, we performed an lMSD analysis for every single lysosomal trajectory. Previous studies have implemented this time-resolved analysis, however either distinguishing only between active and passive transport [54,55] or without afterwards analysing collectively the trajectory parts of each motion category [9,18,19].

**Figure 4.**
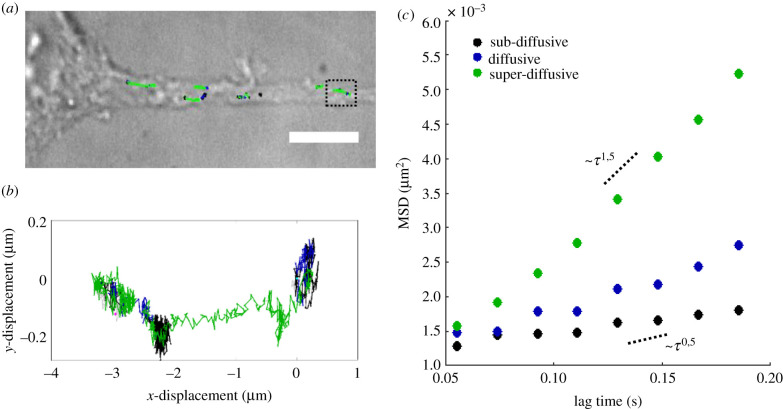
Time-resolved characterization of motion, based on local MSD analysis. (*a*) Bright-field image of a differentiated PC12 cell with its neurite aligned along the patterned laminin line. Recorded trajectories of lysosomes are overlaid and colour coded based on the type of motion (sub-diffusive for *α* ≤ 0.9, diffusive for 0.9 ≤ *α* ≤ 1.1 and super-diffusive for *α* ≥ 1.1). The *α*-value was determined using the local MSD analysis. Scale bar equals 10 μm. (*b*) Close-up of the trajectory indicated by the black dotted square in (*a*). (*c*) MSD of the three motion modes, from the parts comprising the trajectory shown in (*b*). The dotted lines indicate the theoretical sub-diffusive (*α* ∼ 0.5) and super-diffusive (*α* ∼ 1.5) MSD curves.

Here, we differentiated among the three modes of lysosomal motion, characterized each trajectory data point and subsequently analysed collectively the trajectory parts of each motion type. Figure 4*a* shows the bright-field image of a differentiated cell, with its neurite aligned along the laminin line, overlaid with recorded trajectories of lysosomes. Figure 4*b* displays the lysosomal trajectory indicated by the dotted black square in figure 4*a*. The trajectory parts are colour coded, indicating either sub-diffusive (black), diffusive (blue) and super-diffusive (green) transport. For each mode of motion in this trajectory, the respective MSD curve is displayed in figure 4*c*. In the same plot, dashed lines indicate theoretical MSD curves of super-diffusion (*α* ∼ 1.5) and sub-diffusion (*α* ∼ 0.5).

### 3.5. Neurite alignment associates with more efficient (sub-/ super-)diffusive transport of lysosomes

**Figure 5.**
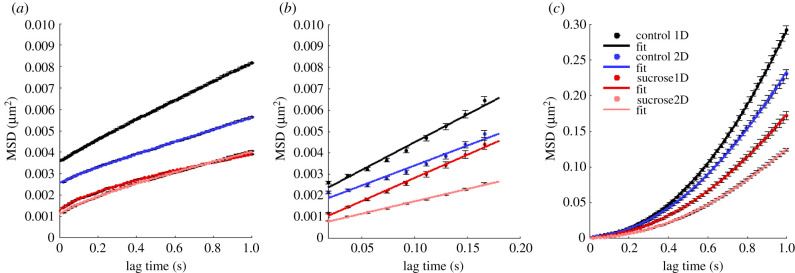
MSD per transport mode. (*a*) Sub-diffusive, (*b*) diffusive and (*c*) super-diffusive MSD plots of the respective lysosomal trajectories data points, as determined using the lMSD analysis. Colour coding indicates lysosomes inside aligned (one-dimensional, 1D) or randomly oriented (two-dimensional, 2D) neurites of differentiated PC12 cells in medium without (control) or with sucrose.

After characterizing every individual trajectory in a time-resolved manner using the lMSD analysis, we analysed collectively all trajectory data points of each transport type. The MSD curves of the sub-diffusive, diffusive and super-diffusive parts of lysosomal trajectories in neurites of differentiated PC12 cells for the four experimental conditions are displayed in figure 5. The sub-diffusive MSD curves were fitted using the power law describing anomalous diffusion (equation (2.5)). To fit the diffusive MSDs, the Brownian motion model was used (equation (2.6)). Lastly, for the super-diffusive MSDs we implemented the model of Brownian motion with drift (equation (2.7)) [14]. The resulting fitting parameters are summarized in electronic supplementary material, table S1.

Lysosomes exhibit a higher (vectorial) MSD inside aligned neurites than in randomly oriented neurites, for all three motion modes studied. This alignment-associated effect appears to be consistent also in the case of the sucrose-induced perturbation, except for the sub-diffusive trajectory modes. As the resulting experimental values indicate (electronic supplementary material, table S1), the diffusion coefficient of the diffusive trajectory modes is higher for lysosomes inside aligned neurites, without or with the presence of the perturbation (0.0065 μm^2^ s^−1^ and 0.0054 μm^2^ s^−1^, respectively), than for lysosomes inside randomly oriented neurites (0.0047 μm^2^ s^−1^ and 0.0029 μm^2^ s^−1^, respectively). Similarly, the drift velocity of the super-diffusive trajectory parts is higher for the lysosomes moving inside the aligned neurites, in the media both without and with sucrose (0.538 μm s^−1^ and 0.397 μm s^−1^, respectively), than the respective values for randomly oriented neurites (0.460 μm s^−1^ and 0.343 μm s^−1^, respectively). These findings are in agreement with the results for the *x*- and *y*-axes presented in figure 3, further confirming the association between neurite alignment and larger lysosomal displacements.

### 3.6. Sucrose accumulation enhances crowding in aligned neurites

The experimental value of the sub-diffusive *α*-exponent is found to be equal to 0.92 and 0.66 for lysosomes inside aligned neurites without and with sucrose, respectively. Thus, the decrease in the sub-diffusive *α*-value, attributed to sucrose accumulation, is approximately equal to 0.26, which is twice the respective decrease observed inside randomly oriented neurites. The decrease in the diffusion coefficient attributed to the incubation of the cells with sucrose is approximately equal to 17% for lysosomes inside aligned neurites, which is almost half of the respective 35% for lysosomes inside randomly oriented neurites. Interestingly, our results indicate a sucrose-associated decrease in the drift velocity of the super-diffusive trajectory modes, approximately equal to 25%, which is the same for lysosomes inside both neurite configurations.

### 3.7. Sucrose-induced lysosomal swelling results in a proportionate decrease in the diffusion coefficient

Previously we found the mean diameter of lysosomes to be equal to 〈*d*_*c*_〉 = 0.82 ± 0.02 μm and 〈*d*_*s*_〉 = 1.15 ± 0.02 μm for differentiated PC12 cells in medium without sucrose (control) versus medium with sucrose, respectively (figure 2*e*). According to the Stokes–Einstein equation [56], the diffusion coefficient *D* of a spherical particle with radius *r*, through a liquid with low Reynolds number, is given by

3.1 $$D=\frac{k_{B}T}{6\pi \eta r},$$

where *k*_*B*_ is the Boltzmann constant, *T* is the temperature and *η* is the viscosity. Assuming that the temperature and viscosity remain constant, we expect the ratio of two diffusion coefficients, *D*_1_ and *D*_2_, of two particles to be analogous to the inverse ratio of their respective diameters, *d*_1_ and *d*_2_ : (*D*_1_/*D*_2_) = *d*_2_/*d*_1_. Thus, supposing there is no difference in the average temperature or viscosity of the cytoplasm between the cells in normal medium and the cells in medium containing sucrose, we expect the average diffusion coefficients and diameters of lysosomes in medium without (control) versus with sucrose to satisfy

3.2 $$\frac{\left\langle d_{s}\right\rangle }{\left\langle d_{c}\right\rangle }=1.4=\frac{\left\langle D_{c}\right\rangle }{\left\langle D_{s}\right\rangle }.$$

The experimental diffusion coefficients of the lysosomes inside cells in normal medium and in sucrose-containing medium, for the two configurations (electronic supplementary material, table S1), yield: (〈*D*_*c*_〉/〈*D*_*s*_〉)_1*D*_ ≈ 1.2 and (〈*D*_*c*_〉/〈*D*_*s*_〉)_2*D*_ ≈ 1.7, a result close to the expected value, with the case of the aligned configuration exhibiting lower deviation from the expected result.

### 3.8. JDD analysis yields the same characteristic values as MSD analysis for diffusive motion modes

The JDD is increasingly used for the characterization of intracellular trajectories [16,55,57]. Thus, as a last step in our analysis, we investigated how the results of these distributions and the characteristic values extracted from their fits relate to the respective ones extracted from the MSD analysis.

**Figure 6.**
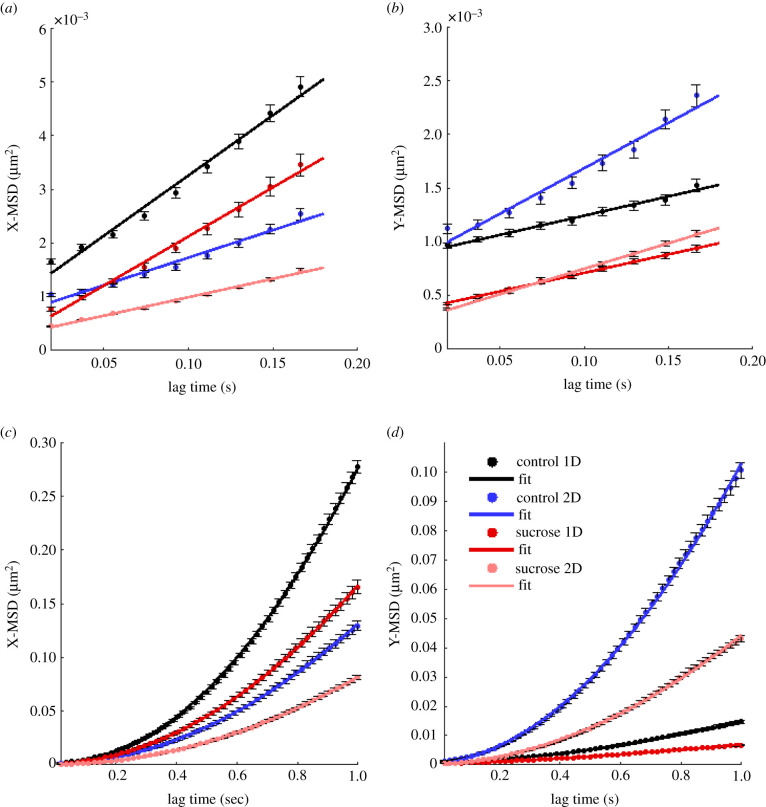
X- and Y-MSDs of diffusive and super-diffusive trajectory parts. MSDs calculated collectively for all diffusive trajectory parts along (*a*) the *x*- and (*b*) *y*-axes. MSDs calculated collectively for all super-diffusive trajectory parts along (*c*) the *x*- and (*d*) *y*-axes. Colour coding indicates data for lysosomes inside aligned (one-dimensional, 1D) and randomly oriented (two-dimensional, 2D) neurites of differentiated PC12 cells in media without (control) or with sucrose. Diffusive MSDs (*a*,*b*) were fitted using equation (2.6). Super-diffusive MSDs (*c*,*d*) were fitted using equation (2.7). The fitting parameters are summarized in electronic supplementary material, table S2.

**Figure 7.**
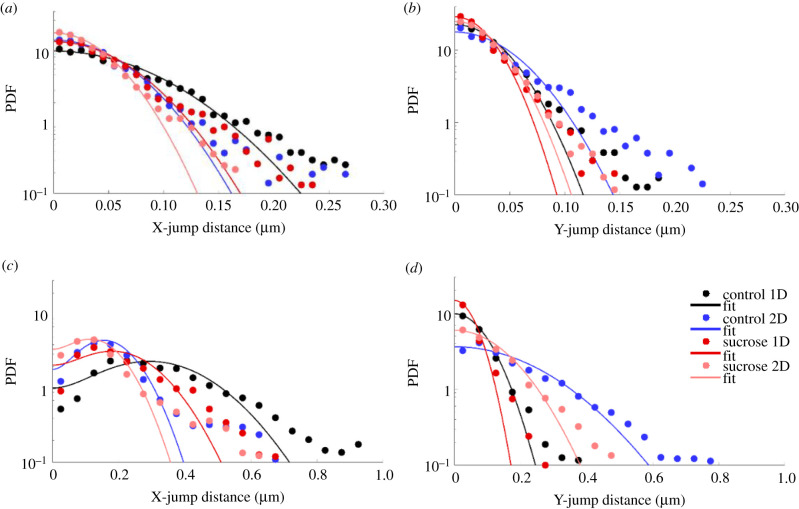
X- and Y-JDD PDFs of diffusive and super-diffusive trajectory parts. JDD PDFs calculated collectively for all diffusive trajectory parts along (*a*) the *x*- and (*b*) *y*-axes, for *τ* = 240.5 ms. JDD PDFs calculated collectively for all super-diffusive trajectory parts along (*c*) the *x*- and (*d*) *y*-axes, for *τ* = 758.5 ms. Colour coding indicates data for lysosomes inside aligned (one-dimensional, 1D) and randomly oriented (two-dimensional, 2D) neurites of differentiated PC12 cells in media without (control) or with sucrose. Diffusive JDD PDFs (*a*,*b*) were fitted using equation (2.9). Super-diffusive JDD PDFs (*c*,*d*) were fitted using equation (2.10). The fitting parameters are summarized in electronic supplementary material, table S3.

In figure 6, the X- and Y-MSD curves of the diffusive and super-diffusive parts of lysosomal trajectories in neurites of differentiated PC12 cells for the four experimental conditions are displayed, along with the fitting curves. The respective PDFs and corresponding fitting curves of the jump distances along the *x*- and *y*-axes are presented in figure 7. Consistent with the MSD, the JDD displacement along the *x*-axis (same as the neurite alignment axis) is larger in the case of the aligned neurites, for conditions both without and with sucrose incubation. Additionally, incubation with sucrose appears to be associated with smaller displacements than in the control case.

All diffusion coefficient and velocity values resulting from the fits of the MSD and JDD curves are summarized in electronic supplementary material, tables S2 and S3. It is remarkable how similar the resulting values of the X- and Y-components of the diffusion coefficient for the diffusive trajectory modes are. The resulting values of the X-component of the drift velocity of the super-diffusive trajectory modes exhibit small differences; the values extracted via fitting the JDD PDFs are systematically smaller than the respective ones derived from the MSD fits. This is of no surprise since, as can be seen, the fitting curves neglect the longer tails. However, the trend observed among the experimental conditions is maintained: in the presence of sucrose the velocity is smaller than the control case, regardless the neurite configuration, and the one-dimensional alignment indicates higher values than the random orientation case, regardless of the presence of the perturbation. The Y-component of the drift velocity of the super-diffusive trajectory modes, estimated via fitting the JDD PDF, deviates from the other values by two orders of magnitude.

## 4. Discussion

Cell survival, growth and conservation of homeostasis rest upon fine tuning and interplay of a plethora of processes. One such vital process is the transport of organelles, proteins or debris within the cellular environment. Malfunctioning intracellular motion is associated with neurodegenerative diseases [1–5], emphasizing the significance of this process for neuronal cells more than other cell types. However, a deeper insight is required to determine whether faulty intracellular transport underlies or gives rise to the pathology of these diseases.

Here, we employ a model system of neuron-like cells [32] to characterize the motion of lysosomes inside their neurites. As the cell shape affects the organization of the cytoskeleton and thereby the intracellular transport, we investigate this effect by guiding the neurites of differentiated PC12 cells towards two distinct geometries: either randomly oriented on a surface or aligned along chemically patterned lines. In parallel, to mimic a pathological cellular phenotype, we perturb the cellular homeostasis via sucrose accumulation and induced lysosome swelling, and detect via motion analysis how its effect varies with the neurite geometry.

The overall MSD and JDD plots of lysosomal trajectories indicate enhanced transport when the neurites are aligned. The length scale of the observed trajectories is small compared with the neurite width or curvature. This means that lysosomes moving inside randomly oriented neurites experience, locally, straight sections similar to what lysosomes inside one-dimensional-aligned neurites encounter. Despite the resemblance in the surrounding environment that lysosomes experience in the span of their trajectories, neurite alignment does influence cytoskeletal organization. An analysis we performed estimating the coherency coefficients of immunofluorescently labelled microtubules and actin filaments (electronic supplementary material, figure S1) suggests that wider neurites have less microtubule coherency (with values ranging between 0.3 and 0.4, as compared with values between 0.6 and 0.75, shown in electronic supplementary material, figure S1, column (iii)). This effect appears to occur when the neurites are allowed to grow randomly on two dimensions, whereas when they align on the one-dimensional lines their width is smaller and more consistent. Moreover, our results suggest that there may be an association with neurite alignment and higher coherency of the actin filaments. Thus, we can assume that the enhanced transport we observe can be attributed to a global rearrangement of the cytoskeletal components resulting from the alignment of the neurites, confirming the hypothesis that the cell shape impacts intracellular transport. Further characterization of the cytoskeletal components in our experimental system is necessary to better investigate the interplay between the cell’s filamentous network and organelle motion.

Additionally, it should be noted that a qualitative inspection of our data (see electronic supplementary material, figure S2) revealed no correlation between lysosomal transport and the positioning of the trajectories within the neurites. It would be interesting, in future experimental work, to investigate and quantitatively characterize whether such a correlation exists and explore whether and how it is affected by varying neurite geometry.

Implementing local MSD analysis, we separately characterize the sub-diffusive, diffusive and super-diffusive transport phases of the recorded lysosomal trajectories [9,18,19]. We find that both diffusive and super-diffusive motion of lysosomes is enhanced inside aligned neurites. In addition, this result is maintained in the case where the homeostasis has been impaired via sucrose incubation, suggesting a global effect of neurite alignment on organelle transport. Our quantitative analysis indicates higher drift velocity for lysosomes inside aligned neurites, an effect which would be very interesting to characterize in more detail in future experimental work by investigating the transport characteristics of motor proteins inside the varying neurite configurations and the subsequent interplay with organelle transport, similarly to previous experiments characterizing granule or RNA motion in cellular protrusions [58,59]. For the sub-diffusive trajectory parts, the apparent motion enhancement is smaller, as seen from the alpha-exponent values of the MSD curve fits, which are slightly larger for lysosomes inside aligned neurites. Our findings complement previous studies, which used lMSD analysis to investigate the effect of cytoskeleton organization on intracellular dynamics of *Dictyostelium discoideum* cells [9,10,17,19], demonstrating the potential of this analysis also for mammalian intracellular organelle motion.

In addition, we confirm that sucrose induces swelling of lysosomes inside differentiated PC12 cells with an associated increase in their average diameter by 0.33 μm, and this leads to a proportionate decrease in their diffusion coefficient, as estimated by fitting the MSD curves of the diffusive trajectory modes. However, the neurite alignment seems to alleviate the sucrose effect in the case of the diffusive motion, resulting in half the respective decrease in the diffusion coefficient of lysosomes inside non-aligned neurites. Our quantitative estimation was based on the assumption that the cytoplasmic viscosity does not change significantly after sucrose treatment, a hypothesis that has been previously experimentally supported [53].

Our results contradict the findings reported in the same article regarding active transport, as we observe a sucrose-associated decrease in the drift velocity of the super-diffusive transport modes, for both neurite configurations. Apart from the fact that we used a different cell type and we studied motion inside the neurites and not in the main cell body, we assume this discrepancy to be associated with the differences in statistics, frame rate during the data acquisition process and the motion analysis performed. Bandyopadhyay *et al*. [53] considered directed translocation for at least 1.5 s (five frames) as active and analysed data from 20 lysosomes, for five cells total. With our high frame rate (52 frames per second) combined with the implementation of lMSD analysis, we have a much more specific discrimination criterion of super-diffusive phases within a trajectory. In addition, the number of cells and lysosomes we studied is much higher, thus providing a statistically significant result.

Furthermore, our results reveal a decrease in the alpha-exponent of the sub-diffusive trajectory modes, suggesting a crowding effect due to sucrose incubation [60,61]. The apparent higher crowding inside aligned neurites as compared with the randomly oriented ones might be associated with the underlying pattern-induced homogeneity of the aligned neurite diameter, which is systematically similar to or smaller than the respective diameters that the randomly oriented neurites have the freedom to adopt. Moreover, we speculate that the contradictory result of previous work by Reverey *et al.* [62] could be attributed to the fact that their work is on pathogenic amoebas, a totally different cell type, which is also highly motile. Although the authors report increased super-diffusive intracellular motion with increasing cytoplasmic crowding, they hypothesize that it is a strategy of this cell type to facilitate particle transport inside such a highly crowded environment [62].

Lastly, the JDD analysis of the diffusive motion modes resulted in values surprisingly close to the respective ones extracted from the MSD analysis. This was not the case for the super-diffusive trajectory parts, where the resulting values deviated significantly from the respective ones estimated using the MSD curve fitting. In future studies, it would be interesting to investigate whether this might be resolved after further discrimination of super-diffusive trajectory parts, with *α* values between 1.1 and approximately 1.7, and active transport parts, with *α* values approximately 2 ± 0.3, as the long tails in the JDD PDFs are neglected in the fits.

In summary, the experiments presented here are the first to quantitatively characterize the motion of functional organelles inside neurites of two different geometries, and compare the effect of sucrose-induced swelling on lysosomal motion, inside each neurite configuration and for each of the three transport modes. We confirm that the dimensionality of the neurites distinctly affects lysosome transport, with a positive correlation between one-dimensional neurite alignment and higher diffusion coefficients and drift velocities of the lysosomal motion modes. Additionally, we show that disruption of homeostasis via sucrose accumulation and induced lysosomal swelling has a larger effect when the motion occurs inside randomly oriented neurites. Future research could expand upon the interplay between organelle transport and other neurite geometries or disease-associated disruptions of the cellular homeostasis.

## Data accessibility

All scripts used in this study are openly accessible through https://github.com/StochasticBiology/boolean-efflux.git. The data are provided in the electronic supplementary material [63]. Data can be made available upon request to the corresponding author.
